# Evaluation of Sentinel Node Biopsy in Locally Advanced Breast Cancer Patients Who Become Clinically Node-Negative after Neoadjuvant Chemotherapy: A Preliminary Study

**DOI:** 10.4061/2011/870263

**Published:** 2011-10-12

**Authors:** Shaji Thomas, Apurva Prakash, Vinay Goyal, Manju Bala Popli, Shilpi Agarwal, Monisha Choudhury

**Affiliations:** ^1^Departments of Surgery and Pathology, Lady Hardinge Medical College, Shaheed Bhagat Singh Marg, New Delhi 110001, India; ^2^Department of Radiological Imaging, Institute of Nuclear Medicine and Allied Sciences, Delhi 110054, India

## Abstract

*Introduction*. Controversy continues over the appropriate timing of sentinel lymph node (SLN) biopsy in locally advanced breast cancer (LABC) patients receiving neoadjuvant chemotherapy. We evaluated the feasibility and accuracy of SLN biopsy in LABC patients with cytology-proven axillary nodal metastasis who become clinically node-negative after neoadjuvant chemotherapy. *Materials*. 30 consecutive patients with LABC, who had become clinically node-negative after 3 cycles of neoadjuvant chemotherapy, were included in the study. They were then subjected to SLN biopsy, axillary lymph node dissection, and breast surgery. *Results*. Sentinel nodes were successfully identified in 26 of the 30 patients, resulting in an identification rate of 86.67%, sensitivity of 83.33%, false negative rate of 20%, negative predictive value of 72.73%, and an overall accuracy of 88.46%. No complications were observed as a result of dye injection. *Conclusions*. SLN biopsy is feasible and safe in LABC patients with cytology-positive nodes who become clinically node-negative after neoadjuvant chemotherapy. Our accuracy rate, identification rate, and false negative rate are comparable to those in node-negative LABC patients. SLN biopsy as a therapeutic option in LABC after neoadjuvant chemotherapy is a promising option which should be further investigated.

## 1. Introduction

Prognosis in patients with breast cancer depends mainly on the extent of lymph node involvement, size of the tumor, and the histological grade of the tumor. Among these factors, axillary lymph node status is regarded as the single best marker of prognosis [1, 2].

For axillary nodal involvement, treatment in the form of level I and II axillary lymph node dissection (ALND) is considered optimum. But it is also associated with a number of complications including self-limiting complaints of numbness (70%), pain (33%), weakness (25%), swelling (24%), and stiffness (15%) which can interfere with daily living in upto 39% of cases. The risk of arm edema varies from 8% to 37% being related to the level of dissection and the number of nodes removed. Axillary vein thrombosis and injury to the motor nerves of axilla are extremely uncommon [2, 3].

Following the introduction of sentinel lymph node (SLN) biopsy for breast cancer, this technique has been widely adopted by cancer centers around the world for node-negative early breast cancer [2]. If the sentinel lymph node (SLN) is negative, the likelihood for other lymph nodes in the axilla to be negative ranges from 95 to 100%. So unnecessary ALND can be avoided in patients with negative axillae, and the associated morbidity of ALND can be reduced [4]. 

Neoadjuvant chemotherapy can reduce tumor size and downstage the primary tumor. Several studies could demonstrate a significant reduction in tumor size and a significant increase in breast-conserving surgery in operable breast cancer [5, 6]. Response to treatment is also an excellent indicator of chemotherapy effectiveness [5, 7–9]. In locally advanced breast cancer (LABC), treatment typically includes neoadjuvant chemotherapy, surgery, and radiation therapy [2]. 

Controversy continues over the appropriate timing of SLN biopsy in LABC patients receiving neoadjuvant chemotherapy as it could offer them the potential benefit of axillary downstaging and avoidance of axillary dissection. Recently, it has been shown that in locally advanced breast cancer, patients with a complete pathologic axillary response had a significantly higher overall survival than patients with residual disease [10]. Their study validated the prognostic stratification of patients with a complete pathological axillary response to neoadjuvant chemotherapy.

The aim of this study was to evaluate the feasibility and accuracy of SLN biopsy in locally advanced breast cancer patients with cytology-proven axillary nodal metastasis who become clinically node-negative after neoadjuvant chemotherapy.

## 2. Materials and Methods

The present prospective observational study was conducted by the Departments of Surgery and Pathology at Lady Hardinge Medical College, New Delhi.

A total of 30 consecutive patients (accrued over a period of 18 months) with cytology/biopsy-proven locally advanced breast cancer LABC (AJCC Stage III) and cytology-proven axillary nodal metastasis, who became clinically node-negative (on clinical examination) after neoadjuvant chemotherapy, were included in the study. Patients who had prior axillary surgery and those with inflammatory breast cancer were excluded from the study. The study was cleared by the Institutional Ethics Committee. Informed consent was taken from all the patients for the planned procedure.

All patients were subjected to a detailed clinical evaluation, routine investigations, and metastatic workup at the time of presentation. Investigations included a complete hemogram, blood sugar, liver function tests, kidney function tests, ECG, echocardiogram, chest X-ray, an ultrasound of the abdomen, and a bone scan. Breast investigations included a mammogram, breast ultrasound, cytology, and trucut biopsy for ER/PR/Her2 status. Breast ultrasound was used to define clinical response of the breast tumor to chemotherapy. 

The patients were given three cycles of neoadjuvant chemotherapy (CAF: cyclophosphamide 600 mg/m^2^, adriamycin 50 mg/m^2^, 5-flurouracil 600 mg/m^2^), and patients who satisfied the inclusion criteria were subjected to sentinel node biopsy, axillary lymph node dissection, and breast surgery. All patients were operated by the same consultant.

After the administration of anesthesia, the planned incision was marked by a marking pencil on the skin of the breast containing tumor. 2 to 5 mL of sterile 1% isosulphan blue (Patent Blue) dye was injected peritumourally (upper outer aspect of tumor) using a syringe and a 22G needle. This was followed by a breast massage for 10 minutes. Incision was made just below the axillary hairline. The incision was planned in such a way as to be included in the mastectomy incision in patients undergoing modified radical mastectomy. Dissection was rapidly done in the axilla to the clavipectoral fascia, and on reaching the fascia, the blue stained lymphatic(s) was carefully identified and traced up to the blue sentinel node(s). Sentinel node(s) was identified by its blue stain. Sentinel node(s) was then harvested before performing the breast surgery. Standard axillary dissection (ALND) was then performed removing level I and II axillary lymph nodes. Lymph nodes removed during ALND were labeled as nonsentinel nodes (NSN). Patients were observed for any immediate or late complications associated with dye injection.

For the purpose of histopathological examination (which included cytokeratin immunohistochemistry), sentinel node and nonsentinel nodes were submitted separately for histopathological analysis. In cases where more than one sentinel node was found, each node was analyzed as sentinel node. 

A standardized set of data was abstracted from each patient. Data collection from all the patients was then analyzed using SPSS statistical software (version 9.0; SPSS Inc, Chicago, ILL, USA).

## 3. Observations and Results

The study group comprised of 30 consecutive patients of locally advanced breast cancer (AJCC Stage III) with cytology-proven axillary lymphadenopathy at presentation who became clinically node-negative (on clinical examination) after completion of three cycles of neoadjuvant chemotherapy. These patients were then subjected to sentinel lymph node biopsy followed by axillary lymph node dissection at the same operation. Out of these 30 patients, 29 patients underwent modified radical mastectomy while one patient had breast conserving surgery done. 

In the present study, the mean age of the patients was 49.45 years (range from 25 to 65 years, median age 45 years). All the patients in the present study were females. Out of the 30 patients, 9 (30%) were premenopausal, while 21 (70%) were postmenopausal. None of the patients had a family history of breast, colon, ovary, or any other cancer. 60% (18/30) of patients had primary tumor in the left breast, while 40% (12/20) patients had tumor in the right breast. The size of the primary tumor varied from 3 cm to 7.5 cm (T2, T3, and T4b).

All the 30 patients had infiltrating ductal cancer, out of which 1 was well differentiated (grade 1), 21 were moderately differentiated (grade 2), and the remaining 8 were poorly differentiated (grade 3). 14 patients were estrogen receptor positive, 12 were progesterone receptor positive, and 11 patients had Her2 overexpression. 

Out of these 30 patients, minimal response (less than 50% reduction in size) to neoadjuvant chemotherapy was seen in 14 patients (46.67%), partial response (more than 50% reduction in size) was seen in 14 patients (46.67%), and complete response was seen in the remaining 2 patients (6.67%). Breast ultrasound was used to define clinical response of the breast tumor to chemotherapy.

Out of the total of 30 patients who underwent SLN biopsy in the present study, the sentinel node was successfully identified in 26 patients. Sentinel node identification rate was 86.67%. The number of sentinel nodes removed per patient ranged from 1 to 4 (1(*n* = 18), 2(*n* = 8), 3(*n* = 3), 4(*n* = 1)). Average number of sentinel node identified per patient was 1.57. In most of the patients, sentinel node identified was 1 in number.

The number of nonsentinel nodes (NSN) identified ranged from 9 to 19. Average number of nonsentinel node identified after ALND was 13.5. Median number of NSN identified was 13. 

Out of the total 26 cases in which a sentinel node was identified, the sentinel node was positive for tumor metastasis in 15 cases, the rest were negative on histopathology. In the 15 cases when the sentinel node was positive for metastasis, the nonsentinel nodes were positive for tumor metastasis in 12 cases. In the rest 3 cases in which the sentinel node was positive, the nonsentinel nodes were found to be negative for tumor metastasis on histopathology (Table 1). It appears that in these 3 patients, the sentinel nodes were the only involved nodes.

Out of the 11 cases when the sentinel node was negative for metastasis, in 8 cases the nonsentinel nodes were also negative for tumor metastasis on histopathology, while in the remaining 3 cases, the nonsentinel nodes were positive for tumor metastasis, thereby accounting for 3 false negatives in the study (Table 1). Out of these 3 patients, 2 had only one non-sentinel node positive for tumor, while the third had 2 non-sentinel nodes showing tumor. It is possible that fibrosis around the involved nodes could have resulted in the false negatives.

Thus in our study, sentinel node dissection was attempted in 30 patients, out of which sentinel nodes were successfully identified in 26 patients, with a sentinel node identification rate of 86.67%. We achieved a sensitivity of 83.33% (15/18), false negative rate of 20% (3/15), a negative predictive value of 72.73% (8/11), and an overall accuracy of 88.46% (23/26).

No complications were observed as a result of dye injection in any of the patients. All of the patients had a bluish green discoloration of the body (especially the face) and observed green-colored urine for 12 to 24 hours after surgery.

## 4. Discussion

Although the impact of resecting axillary lymph nodes on survival is currently a subject of controversy, accurate assessment of axillary nodal status provides the most important prognostic information for patients with primary breast cancer. It also directs selection of adjuvant systemic therapy and reduces the risk of regional recurrence of breast cancer in the axilla [2, 11, 12]. 

 Following the introduction of sentinel lymph node (SLN) biopsy for early breast cancer, this technique has been widely adopted by cancer centers around the world. If the SLN is negative, the likelihood for other lymph nodes in the axilla to be negative ranges from 95 to 100%. So unnecessary axillary lymph node dissection (ALND) can be avoided, and its attendant morbidity can be reduced, in many patients with small breast cancers and negative axillae [4, 13–16].

Most of the reported experience with SLN biopsy includes patients with clinical stage T1-2 N0 [17]. Locally advanced breast cancer was also considered as one of the contraindications. However, recent studies have now shown that SLN biopsy can be considered if axillary lymph nodes are negative for metastases even in locally advanced breast cancer [10, 18, 19]. 

Neoadjuvant chemotherapy has become the standard of care for the treatment of patients with locally advanced breast cancer and has also been prospectively evaluated in patients with earlier-stage disease [20–23]. Neoadjuvant chemotherapy allows for individual *in vivo* assessment of primary tumor and metastatic lymph node response to chemotherapy. In addition, although chemotherapy is primarily thought of as important in eradicating occult distant disease, it can have a significant effect on locoregional disease as well. Tumor downstaging with neoadjuvant chemotherapy can convert inoperable disease to operable disease and can allow breast-conserving surgery in patients for whom mastectomy is initially the only option for control of locoregional disease [23–25].

During their study in locally advanced cases, Kuerer et al. concluded that neoadjuvant chemotherapy can completely clear the axilla of microscopic disease before surgery, and occult metastases (Isolated Tumor Cells-AJCC pN0(I+)) were found in only 10% of patients with a histologically negative axilla (AJCC pN0: No regional lymph node metastasis histologically). The results of their study have implications for the potential use of sentinel lymph node biopsy as an alternative to axillary dissection in patients treated with neoadjuvant chemotherapy. Their finding that only 10% of patients with complete axillary conversion (histologically negative axilla) have occult nodal metastases suggests that SLN biopsy may be appropriate in patients whose disease is downstaged with neoadjuvant chemotherapy [9].

Cox et al. [10] reported on a series of 89 patients with locally advanced breast cancer subjected to SLN biopsy before neoadjuvant chemotherapy. 27% of their patients had a complete pathologic axillary response; these patients had a significantly higher overall survival than patients with residual disease. Their study validated the prognostic stratification of patients with a complete pathological axillary response to neoadjuvant chemotherapy.

During the last few years, there have been a number of clinical trials on the effectiveness and role of SLN biopsy in patients after preoperative chemotherapy, mainly in early-stage breast cancer with negative nodes [26–28]. 

A retrospective analysis of 428 of 2,365 patients in the NSABP 27 trial who received chemotherapy followed by sentinel node biopsy and an axillary dissection was done [26]. 2,411 patients were randomly assigned to NSABP Protocol B-27. In the 2,365 patients (98.1%) for whom operative and pathology reports were available, there were 428 (18.1%) who had lymphatic mapping and for whom an attempt was made to identify and remove a sentinel node. There were significant differences in the distribution of some of the patient and tumor characteristics between the group of patients who had an SLN biopsy attempted and the group of 1,937 patients (81.9%) who did not. Patients in whom an SLN biopsy was attempted had smaller tumors and clinically uninvolved axillary nodes and are more likely to be lumpectomy candidates. Of the 428 patients in whom lymphatic mapping was attempted, at least one sentinel node was identified and removed in 363. Of the 363 patients in whom at least one sentinel node was identified and removed, 20 patients (5.5%) did not have the required axillary node dissection, leaving 343 patients in whom the accuracy of the sentinel node in correctly staging the axilla could be assessed. Because SLN biopsy was not mandated in the study, there was no predefined protocol dictating the method of lymphatic mapping or the approach to SLN biopsy. In the majority of the cases, nodal positivity was determined by hematoxylin and eosin staining only. However, in a handful of cases, additional immunohistochemical staining was performed to further evaluate the status of sentinel nodes. The analysis of these cases demonstrated an 85% sentinel node identification rate and a false-negative rate of 11%, which are similar to those observed in patients undergoing an initial sentinel node biopsy during the same period [26]. 

Xing et al. in 2006 [27] conducted a meta-analysis of twenty-one studies (total of 1273 patients) that examined the results of SLN biopsy after chemotherapy. The sensitivity of SLN biopsy in the individual studies ranged from 67 to 100 percent, the negative predictive value ranged from 56 to 100 percent, and the overall accuracy ranged from 77 to 100 percent. However, the majority of patients in these studies had stage II breast cancer with negative axillary nodes at presentation. 

The ongoing ACOSOG Z1071 trial “A Phase II study of sentinel lymph node surgery and axillary lymph node dissection following neoadjuvant chemotherapy in women with stage II-IIIB node-positive breast cancer” attempts to determine the false negative rate for sentinel lymph node (SLN) surgery in women with node-positive breast cancer who have completed or plan to undergo neoadjuvant chemotherapy. However, this study includes both stage II and III breast cancer and also includes patients who are node positive after completing the chemotherapy. The trial is expected to be completed by end 2013 [28].

Studies on the feasibility and accuracy of SLN biopsy after preoperative chemotherapy in locally advanced breast cancer patients with documented axillary metastasis are few and the results are inconclusive. 

Shen et al. [29] studied 69 patients with cytology-confirmed axillary metastasis who underwent SLN biopsy after chemotherapy. However, out of these, only 23 were LABC (AJCC stage III). The overall SLN identification rate was 92.8%, and a false negative rate of 25%. They concluded that the status of the SLN cannot be used as a reliable indicator of the presence or absence of residual disease in the axilla in this patient population. Newman et al. [30] evaluated 54 breast cancer patients with biopsy-proven axillary nodal metastasis. The SLN identification rate after delivery of neoadjuvant chemotherapy was 98%, with a false negative rate of 8.6%. They concluded that SLN biopsy after neoadjuvant chemotherapy in patients with documented nodal disease at presentation accurately identified cases that may have been downstaged to node-negative status and can spare this subset of patients from the morbidity of an ALND. Another recent study from Korea [31] concluded that SLN identification rate, but not accuracy, is significantly decreased after preoperative chemotherapy in axillary node-positive breast cancer patients. They also suggested that for patients who achieve complete axillary clearance by chemotherapy, SLN biopsy could replace ALND. 

In the present study, although having a small sample size, we have showed that SLN biopsy is feasible and safe in locally advanced carcinoma breast who become clinically node-negative after neoadjuvant chemotherapy. Our accuracy rate, identification rate, and false negative rate are comparable to reports in the literature in node-negative LABC patients after chemotherapy. Blue dye method is a safe procedure and none of the patients developed any complications of the dye injection.

LABC consists of a heterogeneous group of patients falling in AJCC stage III. Having advanced stage disease, they have a poor prognosis. However, even in this group, the subgroup of LABC patients in whom there is a complete axillary response to neoadjuvant chemotherapy have a good prognosis—having shown to have a significantly higher overall survival than patients with residual disease [9]. Identification of this subgroup of LABC patients can help target treatment modalities for improved outcomes.

Most of the studies in the literature on SLN biopsy after neoadjuvant chemotherapy involved earlier stage disease, smaller tumors, with negative nodes at presentation. Our study focuses on the likely feasibility and role of SLN biopsy in this good prognosis subgroup of LABC patients with complete axillary response to neoadjuvant chemotherapy. Additional studies are needed to answer the on-going debate regarding optimal treatment of the axilla in LABC patients who are rendered clinically node-negative after neoadjuvant treatment. Following neoadjuvant therapy, accurate evaluation of the axilla by SLN biopsy is feasible in these patients. Sentinel lymph node biopsy as a therapeutic option in locally advanced breast cancer patients who become clinically node-negative after neoadjuvant chemotherapy is a promising option that can spare axillary dissection and its morbidity, and which should be further investigated.

## Figures and Tables

**Table 1:** Tumor metastasis in sentinel and nonsentinel lymph nodes in patients after neoadjuvant chemotherapy when sentinel nodes were successfully identified (*n* = 26).

	Non-sentinel node with metastasis	Non-sentinel node without metastasis	Total
Sentinel node with metastasis	12	3	15
Sentinel node without metastasis	3	8	11

Total	15	11	26

## References

[1] Sainsbury RC, Russel RCG, Williams N S, Bulstrode CJS (2004). The breast. *Bailey and Love's Short Practice of Surgery*.

[2] Burnstein HJ, Harris JR, Morrow M, Devita VT, Lawrence TS, Rosenberg SA (2008). Malignant tumors of the breast. *Cancer: Principles and Practice of Oncology*.

[3] Warmuth MA, Bowen G, Prosnitz LR (1998). Complications of axillary lymph node dissection for carcinoma of the breast: a report based on a patient survey. *Cancer*.

[4] Peintinger F, Reitsamer R, Stranzl H, Ralph G (2003). Comparison of quality of life and arm complaints after axillary lymph node dissection vs sentinel lymph node biopsy in breast cancer patients. *British Journal of Cancer*.

[5] Fisher B, Bryant J, Wolmark N (1998). Effect of preoperative chemotherapy on the outcome of women with operable breast cancer. *Journal of Clinical Oncology*.

[6] Van der Hage JA, Van de Velde CJH, Julien JP, Tubiana-Hulin M, Vandervelden C, Duchateau L (2001). Preoperative chemotherapy in primary operable breast cancer: results from the European Organization for research and treatment of cancer trial 10902. *Journal of Clinical Oncology*.

[7] Fisher B, Brown A, Mamounas E (1997). Effect of preoperative chemotherapy on local-regional disease in women with operable breast cancer: findings from national surgical adjuvant breast and bowel project B-18. *Journal of Clinical Oncology*.

[8] Kuerer HM, Newman LA, Smith TL (1999). Clinical course of breast cancer patients with complete pathologic primary tumor and axillary lymph node response to doxorubicin-based neoadjuvant chemotherapy. *Journal of Clinical Oncology*.

[9] Kuerer HM, Sahin AA, Hunt KK (1999). Incidence and impact of documented eradication of breast cancer axillary lymph node metastases before surgery in patients treated with neoadjuvant chemotherapy. *Annals of Surgery*.

[10] Cox CE, Cox JM, White LB (2006). Sentinel node biopsy before neoadjuvant chemotherapy for determining axillary status and treatment prognosis in locally advanced breast cancer. *Annals of Surgical Oncology*.

[11] NIH Consensus Conference (1991). Treatment of early stage breast cancer. *Journal of the American Medical Association*.

[12] Hsueh EC, Hansen N, Giuliano AE (2000). Intraoperative lymphatic mapping and sentinel lymph node dissection in breast cancer. *Ca-A Cancer Journal for Clinicians*.

[13] Giuliano AE, Haigh PI, Brennan MB (2000). Prospective observational study of sentinel lymphadenectomy without further axillary dissection in patients with sentinel node-negative breast cancer. *Journal of Clinical Oncology*.

[14] Schrenk P, Rieger R, Shamiyeh A, Wayand W (2000). Morbidity following sentinel lymph node biopsy versus axillary lymph node dissection for patients with breast carcinoma. *Cancer*.

[15] Haid A, Kuehn T, Konstantiniuk P (2002). Shoulder-Arm morbidity following axillary dissection and sentinel node only biopsy for breast cancer. *European Journal of Surgical Oncology*.

[16] Swenson KK, Nissen MJ, Ceronsky C, Swenson L, Lee MW, Tuttle TM (2002). Comparison of side effects between sentinel lymph node and axillary lymph node dissection for breast cancer. *Annals of Surgical Oncology*.

[17] Cody HS (2001). Clinical aspects of sentinel node biopsy. *Breast Cancer Research*.

[18] Schrenk P, Tausch C, Wölfl S, Bogner S, Fridrik M, Wayand W (2008). Sentinel node mapping performed before preoperative chemotherapy may avoid axillary dissection in breast cancer patients with negative or micrometastatic sentinel nodes. *American Journal of Surgery*.

[19] Grube BJ, Christy CJ, Black D (2008). Breast sentinel lymph node dissection before preoperative chemotherapy. *Archives of Surgery*.

[20] Hortobagyi GN, Blumenschein GR, Spanos W (1983). Multimodal treatment of locoregionally advanced breast cancer. *Cancer*.

[21] Lippman ME, Sorace RA, Bagley CS (1986). Treatment of locally advanced breast cancer using primary induction chemotherapy with hormonal synchronization followed by radiation therapy with or without debulking surgery. *NCI Monographs*.

[22] Mamounas EP (1998). Overview of national surgical adjuvant breast project neoadjuvant chemotherapy studies. *Seminars in Oncology*.

[23] Singletary SE, McNeese MD, Hortobagyi GN (1992). Feasibility of breast-conservation surgery after induction chemotherapy for locally advanced breast carcinoma. *Cancer*.

[24] Schwartz GF, Birchansky CA, Komarnicky LT (1994). Induction chemotherapy followed by breast conservation for locally advanced carcinoma of the breast. *Cancer*.

[25] Bonadonna G, Valagussa P, Brambilla C (1998). Primary chemotherapy in operable breast cancer: eight-year experience at the Milan Cancer Institute. *Journal of Clinical Oncology*.

[26] Mamounas EP (2003). Sentinel lymph node biopsy after neoadjuvant systemic therapy. *Surgical Clinics of North America*.

[27] Xing Y, Foy M, Cox DD, Kuerer HM, Hunt KK, Cormier JN (2006). Meta-analysis of sentinel lymph node biopsy after preoperative chemotherapy in patients with breast cancer. *British Journal of Surgery*.

[28] National Cancer Institute at the National Institutes of Health http://www.cancer.gov/clinicaltrials/search/view?cdrid=640100&version=healthprofessional#EntryCriteria_CDR0000640100.

[29] Shen J, Gilcrease MZ, Babiera GV (2007). Feasibility and accuracy of sentinel lymph node biopsy after preoperative chemotherapy in breast cancer patients with documented axillary metastases. *Cancer*.

[30] Newman EA, Sabel MS, Nees AV (2007). Sentinel lymph node biopsy performed after neoadjuvant chemotherapy is accurate in patients with documented node-positive breast cancer at presentation. *Annals of Surgical Oncology*.

[31] Lee S, Kim EY, Kang SH (2007). Sentinel node identification rate, but not accuracy, is significantly decreased after pre-operative chemotherapy in axillary node-positive breast cancer patients. *Breast Cancer Research and Treatment*.

